# Data Harmonization Methods and Data Quality Assessment for Integrating Routinely Collected Data Across Multiple Sources: A Systematic Review

**DOI:** 10.63116/AH.000000007

**Published:** 2026-10-21

**Authors:** James M. Kariuki, Anisa Saleh, Chinedu Aniekwe, Mayer Antoine, Neranga Liyanaarachchige, Lisa A. Murie, Daniel H. Rosen, Ronald Cornet

**Affiliations:** 1 Division of Global HIV and TB, Centers for Disease Control and Prevention; 2 School of Public Health, Boston University; 3 Public Health Program, University of Suffolk-UNICAF; 4 Division of Workforce Development, Centers for Disease Control and Prevention; 5 Sri Lanka College of Health Informatics; 6 Public health informatics, Independent Researcher; 7 Amsterdam UMC - University of Amsterdam, Medical Informatics; and Amsterdam Public Health, Methodology & Digital Health

**Keywords:** digital health, data harmonization, data integration, standardized terminologies, interoperability, data quality, routinely collected health data, health information systems

## Abstract

**Background:**

Integrating routinely collected health data across heterogeneous systems is essential for improving patient care, disease surveillance, and programmatic decision-making. However, heterogeneity in data formats, coding systems, and semantic definitions create challenges affecting consistent data interpretation and reuse across systems. Despite its importance, harmonization methods and their relationship to data quality have not been systematically examined. This review analyzes harmonization approaches, reported data quality attributes, associated benefits, and relationships among them.

**Methods:**

We conducted a systematic review following Preferred Reporting Items for Systematic Reviews and Meta-Analyses guidelines. PubMed, Scopus, CINAHL, and ProQuest were searched for studies describing harmonization of routinely collected health data, integrate data from multiple digital systems and reported data quality attributes. Covidence software facilitated screening, full-text review, and data extraction. Descriptive statistics summarized study characteristics, and Spearman correlations assessed associations among number of harmonization methods used, number of systems used, and number of data quality attributes reported.

**Results:**

Of 2,309 records, 69 studies met inclusion criteria. Most (88%) were published between 2013 and 2024, predominantly in high-income countries (93%), with the United States accounting for the majority (64%). Harmonization approaches grouped into 6 categories: established data standards; national/global standards; database/schema mapping; extract-transform-load standardization; manual terminology mapping; and local data dictionaries. Established data standards were most common (40 studies [58%]), followed by national/global standards (26 studies [38%]) and database/schema mapping (20 studies [29%]). Most studies applied 1 or 2 methods (58 [84%]), while 11 (16%) used 3 methods. Frequently reported data quality attributes included completeness (43 [62%]), accuracy (29 [42%]), and timeliness (24 [35%]), while relevance (2 [3%]) and usefulness (1 [1%]) were rarely reported. Overall, 46 studies (67%) reported at least 1 benefit. Data reuse and reduced work burden each reported by 20 studies (29%), and improved decision-making in 18 studies (26%). Correlations indicated negligible associations between number of harmonization methods and number of systems (ρ = 0.028) or number of data quality attributes (ρ = 0.074).

**Conclusions:**

Data element harmonization is essential for integrating heterogeneous health data and enabling interoperability. Standardized approaches dominate, though manual and localized methods remain relevant where global standards are not fully adopted. More consistent reporting of harmonization practices, inclusion of underreported data quality attributes, and examination of contextual factors are needed to strengthen global evidence.

## Introduction

Availability of accurate, complete, timely, and usable data is essential for high-quality health care in every society. Over the past 2 decades, advances in information technology and automation have paved the way for health information systems (HISs) to evolve, meeting the growing demand for routine collection, management, and use of clinical patient data.^1^^,^^2^ These routinely collected clinical and administrative data support decision-making for providers, health managers, and policymakers on patient care, disease surveillance, and health programs.^3^^,^^4^ In most health care settings, patient data are collected and managed in diverse HISs and often spread across multiple providers or organizations. This necessitates integrating these data to provide a holistic view of patient-centered care, continuity of care, disease surveillance, program performance, and care quality indicators.^1–3^^,^^5–9^ Digital health system adoption and interoperability efforts promise the availability of quality data that are crucial for planning interventions, better patient outcomes, resource allocation, public health, and program improvement for many countries and global organizations.^3^^,^^4^^,^^7^^,^^10^^,^^11^

The ability to share and combine heterogeneous data from different sources increases the utility of these data to make decisions at different levels of the health system.^12–15^ Interoperability is the ability of HISs to exchange data, interpret their meaning consistently, and use these for intended purposes. Data element standardization and harmonization are key to contextualizing data and enabling interoperability. Standardization is the process of transforming data elements into common formats, structures, and coding systems to ensure consistency across datasets.^16^^,^^17^ Data harmonization builds on data standardization by aligning data elements with shared semantic definitions, enabling meaningful use across heterogeneous data sources.^18–21^ In this study, we used the term data element harmonization to encompass both standardization and harmonization processes unless otherwise specified.

Data element harmonization is foundational in defining characteristics of data collected and facilitates retrieval, transformation, management, exchange, integration, analysis, and utilization of data across HISs and programs at each stage of the data life cycle.^16^^,^^19^^,^^22^^,^^23^ Ideally, data element harmonization should be planned before data collection. However, most real-world situations integrate data already collected in diverse information systems, some predating current standards, which present operational, representational, and computerization challenges.^12^^,^^24–27^ In most cases, systems are designed to meet specific needs and use diverse programming and database technologies, requiring more effort to align and integrate data. In addition, these systems may have varying data models, data formats, reference ranges, coding systems, validation logic, and data element definitions, affecting the ability to find, access, and integrate data. The data element harmonization process includes utilizing common data models, standardized terminologies, mapping or crosswalks, and alignment of formats, reference ranges, and units of measure. Despite its importance, various harmonization methods are often applied and inconsistently reported, limiting cross-study comparison for best practices.^27^^,^^28^

Assessment of data quality attributes, such as completeness, consistency, accuracy, validity, timeliness, and relevance, is necessary to determine if integrated data are fit for use.^29^^,^^30^ Studies often assert that data harmonization supports data quality and yields benefits such as improved data comparability, enhanced analytic validity, increased efficiency of data reuse, and stronger support for decision-making.^7^^,^^18^^,^^20^^,^^22^^,^^26^^,^^31^ However, the nature of these benefits is often described narratively, with limited synthesis of harmonization methods or data quality attributes assessed. In addition, the relationship between the complexity of data element harmonization (eg, the number of harmonization methods used), the number of data source systems, and assessment of data quality attributes used to support data integration has not been systematically examined.

To our knowledge, no systematic review has comprehensively examined (*a*) the harmonization methods used to integrate routinely collected data from multiple sources, (*b*) the data quality attributes assessed following data element harmonization, (*c*) reported benefits, and (*d*) associations between harmonization methods, the number of data systems involved, and the scope of data quality assessment. Therefore, we conducted a systematic review to synthesize published articles to answer the following questions:

- What data harmonization methods are commonly used to facilitate data integration for routinely collected data from multiple sources?
- Which data quality attributes are most frequently assessed or reported following data element harmonization between different data sources?
- What benefits are commonly reported as a result of integrating data using harmonized data elements?
- What is the nature and strength of the association between the number of methods used to harmonize data elements and (*a*) the number of systems used and (*b*) the number of data quality attributes reported?

## Methods

We conducted a systematic literature review of published articles that used data element harmonization to facilitate data integration of routinely collected health data across different data sources. The review was conducted and reported in accordance with the Preferred Reporting Items for Systematic Reviews and Meta-Analyses guidelines. Covidence, a Cochrane toolkit, was used for screening titles and abstracts, reviewing full text, and extracting data, while Python version 3 in Jupyter notebooks was used for statistical analysis.

### Search Methods

We identified studies by searching PubMed, Scopus, CINAHL, and ProQuest electronic databases. Our primary search used a combination of keywords, synonyms, and related terms for data integration, data elements, digital health sources, and data quality attributes, as shown in Table 1. The search strategy focused on the following 4 groups: data integration, data elements, digital health, and data quality. In the first step, synonyms within each group were combined using “OR.” In the second step, the results of group 1 (data integration) were combined using OR with the results from group 2 (data elements). The results of these 3 searches in steps 1 and 2 were combined using “AND.” The exact search terms used in each electronic database and the search dates can be found in Supplement 1.

**Table 1.** Search Strategy Text Words and Synonyms (N = 69)

**1. Interoperability**			
**Data integration**	**Data elements**	**2. Digital health (sources)**	**3. Data quality outcomes**
Data integration	Data element (metadata)	Digital health	Data quality
Interoperability	Standardized terminology	eHealth	Data completeness
Health information exchange	Metadata management	Electronic medical records	Data accuracy
Data exchange	Clinical concepts	Health management information system	Data consistency
Electronic reporting	Minimum dataset	Laboratory information systems	Data timeliness
Clinical data sharing	Core data elements	Pharmacy information systems	Information quality
Data aggregation	Indicator definitions	Electronic data	
Automated reporting	Data standardization	Electronic health records	
	Quality measures	Computerized clinical records	
		Data repository	
		Data warehouse	
		Database	

The database search was conducted by JK to identify studies in PubMed, Scopus, CINAHL, and ProQuest electronic databases. Results of each database search were imported into the Covidence systematic review software, and duplicate studies were removed.

### Inclusion and Exclusion Criteria

We included original, peer-reviewed articles published in English that met all of the following criteria: (1) described digital health applications used for routine health services; (2) involved user-defined patient care data elements, data standards, or a process for harmonizing data elements to facilitate data integration between 2 or more routine electronic/digital data sources; and (3) evaluated or assessed the integration process using data quality attributes such as completeness, accuracy, consistency, timeliness, availability, or validity. We excluded review articles, perspectives, letters to the editor, studies focused solely on developing data quality frameworks without assessment, and studies not focused on routine health data collection.

### Abstract and Title Screening

Two reviewers independently screened each title and abstract in Covidence to decide whether to include or exclude the study for full-text review. JK reviewed all abstracts, and CA, NL, AS, MA, LM, and RC served as second reviewers. Covidence logged conflicts and facilitated resolution within the platform. Disagreements were resolved by discussing the conflicting article to reach a consensus between the 2 reviewers. JK downloaded the full-text articles for the studies identified and added them to Covidence.

### Full-Text Review

Two reviewers independently reviewed each article and decided whether to include or exclude it for data extraction. JK reviewed all full-text articles, and AS, CA, MA, NL, and LM served as the second reviewers. In case of disagreement, the 2 reviewers met to review the conflicting article and reach consensus.

Inter-rater reliability was assessed for abstract screening and full-text review stages using Cohen kappa (κ), calculated automatically by Covidence based on binary inclusion decisions (include vs exclude) before conflict resolution. We interpreted κ values using established agreement criteria as follows: κ < 0.00 (poor agreement), 0.00-0.20 (slight agreement), 0.21-0.40 (fair agreement), 0.41-0.60 (moderate agreement), 0.61-0.80 (substantial agreement), and 0.81-1.00 (almost perfect agreement).^32^

### Data Collection Process

Two assessors initially independently completed data extraction for 10 articles to validate the extraction template (Supplement 2). The data extraction template included information on first author, title, publication year, article source, country, type of study, study aim, disease or program area, types of systems used, methods used to harmonize data elements, data quality attribute outcomes, and benefits reported. JK extracted data using the data extraction template from the remaining selected articles, and another reviewer verified the correctness of the extraction for each article.

We used an open coding approach to identify and categorize data element harmonization methods. The following categories emerged during the full-text review and were incorporated into the extraction template (Supplement 2):

- *Manual terminology mapping:* Manual alignment of data elements in a system or dataset to standardized terminologies or a common data model by assigning standard codes (eg, mapping local lab names to Logical Observation Identifiers Names and Codes (LOINC) in a crosswalk).^17^^,^^20^^,^^33^
- *ETL-based standardization:* Automated transformation of source data elements into consistent formats, structures, and representations using scripts or tools applied consistently across systems.^17^^,^^26^^,^^31^
- *National/global standards:* Harmonizing data elements to published national or global implementation guides or profiles, using manual or automated data elements mapping within the system (eg, mapping to the US Fast Healthcare Interoperability Resources [FHIR] Core).^3^^,^^5^
- *Established data standards:* Using established internationally recognized terminologies and interoperability specifications within the system, such as *International Classification of Diseases* (ICD), LOINC, SNOMED CT, and Health Level Seven Fast Healthcare Interoperability Resources (HL7 FHIR), to standardize and exchange data.^17^^,^^34^^,^^35^
- *Database/schema variable mapping*: Mapping data elements in the source system to corresponding variables, data types, and formats in the destination database schema or dataset.^3^^,^^17^^,^^22^^,^^36^
- *Local data dictionary*: Using locally defined data dictionaries or data elements documentation to standardize naming, definitions, permissible values, and formats across systems or datasets to support data integration.^3^^,^^22^^,^^26^^,^^31^

### Analysis

Extracted data were exported from Covidence into a CSV file for analysis using Python version 3 in Jupyter notebooks. The data were supplemented with economic categories for the countries where studies were conducted, using the World Bank country income categories.^37^ We tabulated the results of each study to provide an organized presentation of key findings. This included information on study characteristics, methods used to harmonize data elements, data quality attributes reported or assessed, and benefits reported. We used visuals, including charts, and tables, to enhance the clarity of the results and facilitate comparison of the methods and outcomes. We summarized the complexity relationship using count-based measures for each study using the number of source systems integrated, the number of data element harmonization methods reported, and the number of data quality attributes reported. Studies were categorized into simple vs complex patterns using the following thresholds: Simple integrations were defined as those with 2 systems and 1–2 methods, while complex integrations comprised >2 systems and harmonization methods. To visualize the co-reporting patterns of data element harmonization methods and systems used for integration, we created a scatter/bubble plot (number of systems used vs harmonization methods; bubble size = data quality attributes). We examined the nature and direction of the association between the number of methods used to harmonize data elements, the number of systems used for integration, and the number of data quality attributes reported using Spearman rank correlation. These analyses were descriptive and reflected co-reporting patterns across articles rather than causal effects.

## Results

Figure 1 illustrates the systematic selection process for the study. An initial search of PubMed, Scopus, CINAHL, and ProQuest electronic databases yielded 2309 articles. After removing 655 duplicates, 1654 articles underwent title and abstract screening, excluding 1483 articles based on predefined exclusion criteria. Full-text review of the remaining 171 articles resulted in 69 articles that met the predefined inclusion criteria for final analysis.

**Figure 1. PRISMA Flowchart with the Results of Study Selection.**
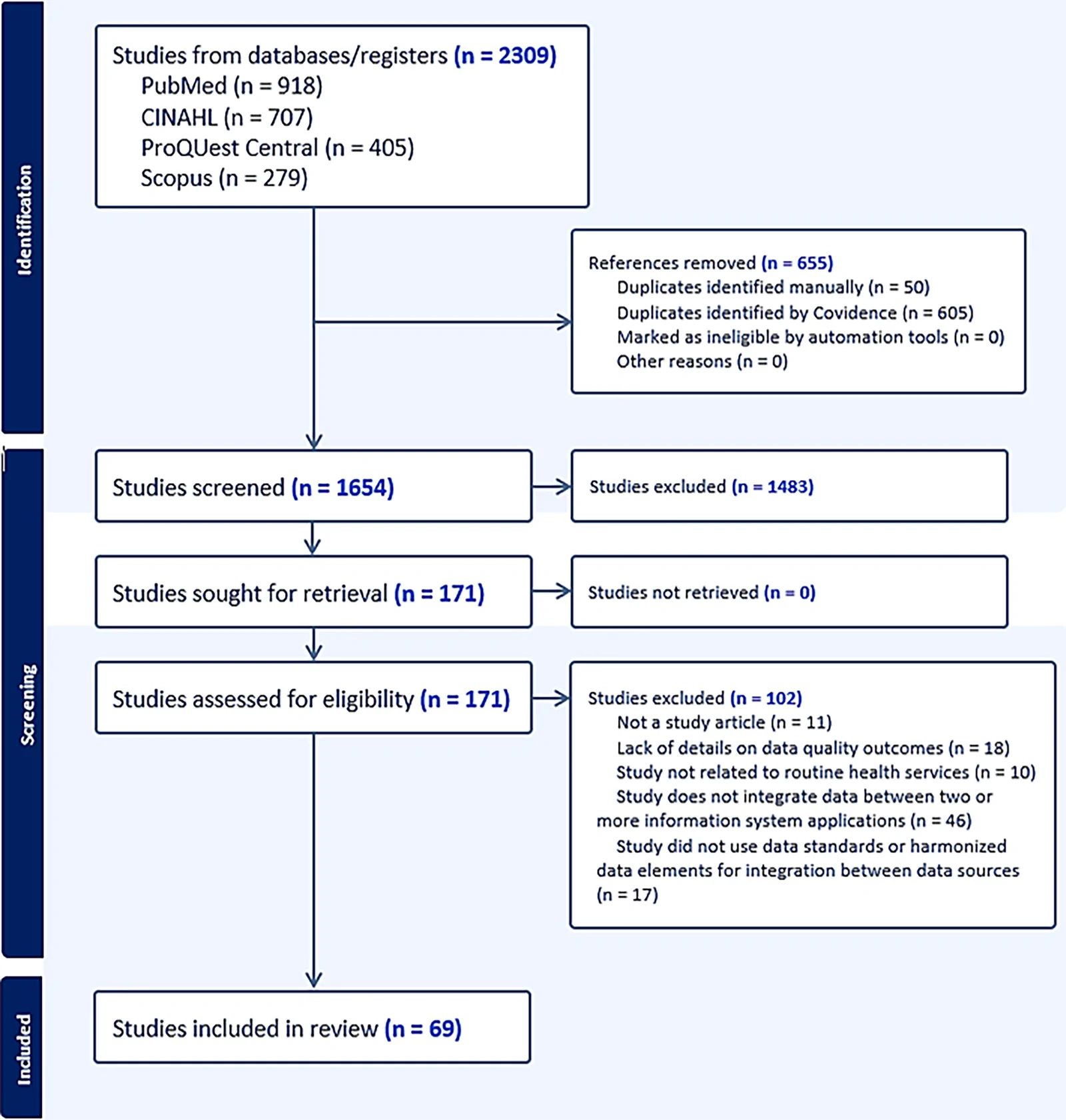
PRISMA, Preferred Reporting Items for Systematic Reviews and Meta-Analyses.

### Inter-Rater Reliability

During title and abstract screening, the proportionate agreement ranged from 0.43 to 0.92, with pairwise Cohen’s κ ranging from −0.17 to 0.39. According to Landis and Koch criteria, most pairs demonstrated fair agreement (κ = 0.21-0.40), with 1 pair (pair 6) showing poor agreement (κ = −0.17), likely because of a small number of items reviewed (n = 7). During the full-text review, proportion agreement ranged from 0.53 to 0.69, and κ values ranged from 0.09 to 0.38, corresponding to the slight to fair agreement. Details on inter-rater reliability across reviewer pairs for both abstract and full-text review stages are provided in Supplement 3.

### Characteristics of the Included Studies

The selected articles for final review were published between 1999 and 2024, as shown in Figure 2, with most of the articles, 61 (88%), published between 2013 and 2024.

**Figure 2.**
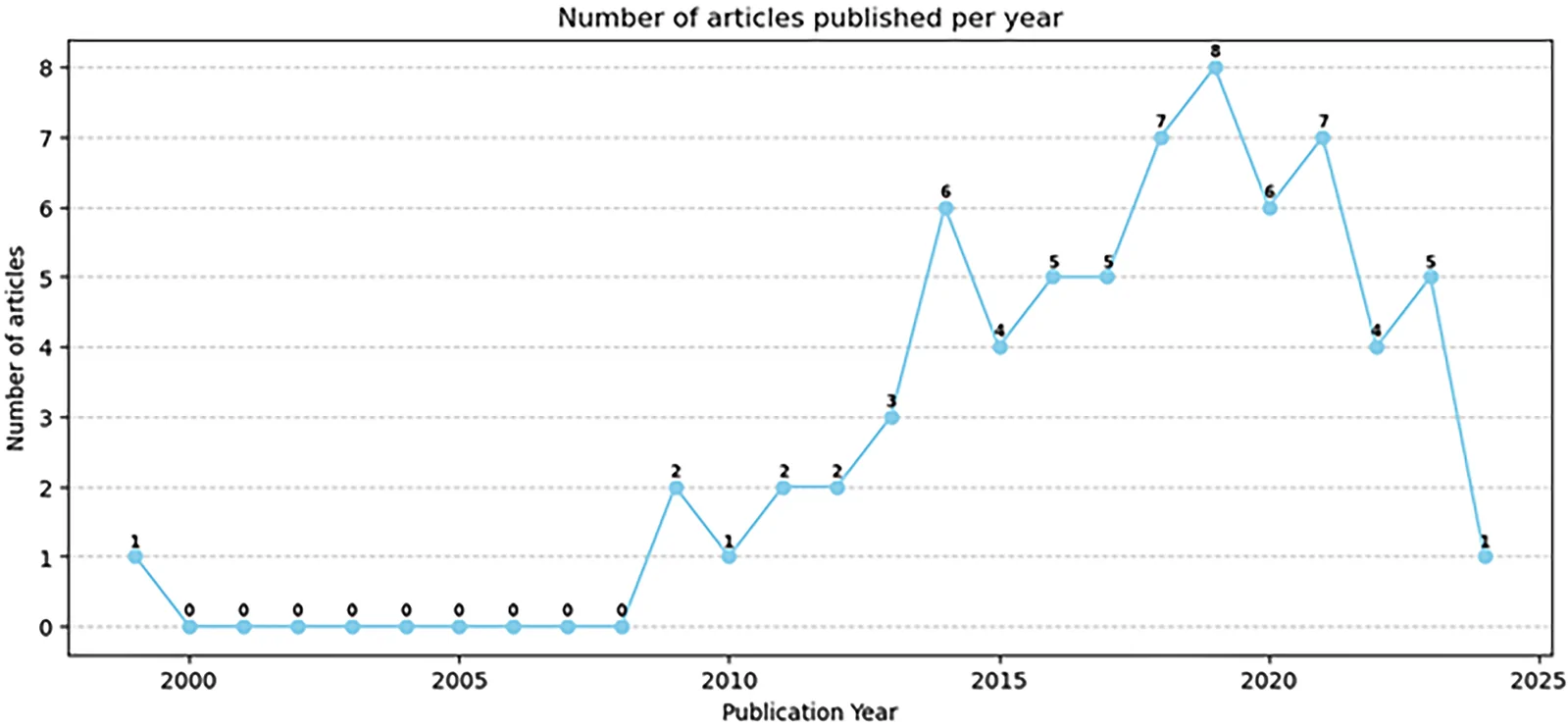
Number of Articles Published Per Year.

Of the 69 articles included in the final analysis, 85% were published in peer-reviewed journals, 13% in conference proceedings, and 1 was a brief report. Most of the articles, 60 (87%), were based on a cross-sectional study method, 3 were case reports, and case series, comparison study, cohort study, controlled before-and-after trial, nonrandomized experimental study, and observational study had 1 article each.

Table 2 summarizes the characteristics of selected study articles and their integration context. Most studies (93%) were conducted in high-income countries, with only 7% from upper- or lower-middle-income settings. Across the world, the studies were conducted in 13 countries, with most (63.8%) concentrated in the United States, followed by Canada, Australia, and Germany with 4 (5.8%) each. Other countries collectively accounted for 9 studies (13%). The most common program areas were immunization (10.1%), notifiable diseases (8.7%), cancer (8.7%), and primary care (8.7%). A wide range of other disease program areas were represented, but most were rare (combined 33.3%). Nearly half of studies integrated 2 systems (46%), while 23% used 3 systems and 25% used 4 systems. Only 6% integrated 5 systems. Integration was dominated by electronic medical/health records (89.9%) and data repositories (65.2%). Other common systems included health information exchanges (HIEs) (23.2%), disease registries (21.7%), and laboratory systems (18.8%). All other system types were rare (combined 11.6%).

**Table 2.** Characteristics of Included Studies and Integration Context (N = 69)

**Study characteristic**	**Category**	**Studies, n (%)**
**Country income category**	High-income	64 (93%)
	Upper-middle income	3 (4%)
	Lower-middle income	2 (3%)
**Country setting**	United States	44 (64%)
	Canada	4 (6%)
	Australia	4 (6%)
	Germany	4 (6%)
	China	2 (6%)
	France	2 (3%)
	Other countries*	9 (13%)
**Disease/program area^**	Immunization - vaccination	7 (10%)
	Notifiable diseases/conditions	6 (9%)
	Cancer	6 (9%)
	Primary care	6 (9%)
	STI/STD	4 (6%)
	Chronic disease	3 (4%)
	Rare diseases	2 (3%)
	Respiratory diseases	2 (3%)
	Neurology	2 (3%)
	Pediatric	2 (3%)
	Clinical care	2 (3%)
	Cardiovascular disease	2 (3%)
	Human immunodeficiency virus (HIV)	2 (3%)
	Mortality Information	2 (3%)
	Mental health	2 (3%)
	All other program areas combined**	23 (33%)
**Number of source systems integrated**	2 systems	32 (46%)
	3 systems	16 (23%)
	4 systems	17 (25%)
	5 systems	4 (6%)
**Types of systems included in the data integration^**	Electronic medical records/electronic health records (EMR/EHR)	62 (90%)
	Data repository (NDR, CDR, DWH, SHR, etc.)	45 (65%)
	Health information exchange (HIE)	16 (23%)
	Disease registry	15 (22%)
	Laboratory information system (LIS)	13 (19%)
	Health management information system (HMIS)	2 (3%)
	Pharmacy information system (PIS)	2 (3%)
	Other types of systems (combined)***	8 (2%)

*Nigeria, Kenya, South Korea, Japan, Sweden, Brazil, Singapore, Finland, and Spain each had 1 article.

**“All other program areas combined” includes 23 categories that each had a count of 1: perinatal health/maternal child health, end-of-life health care, communicable diseases, care of the disabled, care of the mentally handicapped, allergy, social welfare, hypertension, basic health care, specialized health care, inpatient service, cardiovascular conditions, ambulatory clinical care, nephrology, home care, e-prescription, notifiable NCDs, emergency care, reportable conditions, post-market drug surveillance, COVID-19, obesity, and electronic lab reporting.

***“Other types of systems (combined)” includes 8 categories that each had a count of 1: survey assessment tool, commercial death data (CDD), US mortality data (SSDI), administrative: claim/insurance, billing system, scheduling system, death records, and surveillance system.

**^**Counts for program area and types of systems included in the data integration may sum to > N because studies reported multiple program areas and types of systems.

### Approaches/Methods Used to Harmonize Data Elements

Six methods were used to harmonize data elements to support data integration in the selected 69 study articles. The most frequently used method was established data standards, appearing in 58% of the articles, followed by national or global standards (38%), database or schema variable mapping (29%), and extract, transform, and load (ETL)-based standardization (22%). Manual terminology mapping and local data dictionary were less common, each reported in 14% of the studies (Table 3A). For the distribution of number of data element harmonization methods in the studies, 43% used 2 methods,^2^^,^^5^^,^^7^^,^^9–11^^,^^16^^,^^24–26^^,^^28^^,^^31^^,^^33^^,^^36^^,^^38–53^ 41% used a single method,^1^^,^^4^^,^^8^^,^^14^^,^^18^^,^^27^^,^^34^^,^^35^^,^^54–73^ and 16% used 3 methods^3^^,^^6^^,^^13^^,^^15^^,^^17^^,^^20–22^^,^^74–76^ (Table 3B).

**Table 3.** Distribution of Number of Harmonization Methods Used and the Breadth of Harmonization with Details of the Methods (N = 69)

**A. Frequency of data harmonization methods used across studies and breadth of harmonization method(s) reported**			
**Data harmonization method**			**No. of articles, n (%)**
Established data standards			40 (58%)
National/global standards			26 (38%)
Database/schema variable mapping			20 (29%)
ETL-based standardization			15 (22%)
Manual terminology mapping			10 (15%)
Local data dictionary			10 (15%)

**B. Breadth of reporting harmonization methods**			
**Methods used per article**	**Number of articles, n (%)**	**Data harmonization method(s) reported**	**No. of articles reporting method(s)**
1 Method	28 (41%)^1^^,^^4^^,^^8^^,^^14^^,^^18^^,^^27^^,^^34^^,^^35^^,^^54–73^	Established data standards	10
		National/global standards	9
		Database/schema variable mapping	4
		Local data dictionary	3
		ETL-based standardization	1
		Manual terminology mapping	1
2 Methods	30 (43%) ^2^^,^^5^^,^^7^^,^^9–11^^,^^16^^,^^24–26^^,^^28^^,^^31^^,^^33^^,^^36^^,^^38–53^	Established data standards; national/global standards	10
		Database/schema variable mapping; established data standards	6
		ETL-based standardization; established data standards	3
		ETL-based standardization; national/global standards	2
		Established data standards; manual terminology mapping	2
		Local data dictionary; manual terminology mapping	1
		Local data dictionary; national/global standards	1
		Database/schema variable mapping; ETL-based standardization	1
		Database/schema variable mapping; national/global standards	1
		Database/schema variable mapping; manual terminology mapping	1
		Established data standards; local data dictionary	1
		Database/schema variable mapping; local data dictionary	1
3 Methods	11 (16%) ^3^^,^^6^^,^^13^^,^^15^^,^^17^^,^^20–22^^,^^74–76^	ETL-based standardization; established data standards; manual terminology Mapping	2
		Database/schema variable mapping; established data standards; manual terminology mapping	2
		Database/schema variable mapping; ETL-based standardization; established data standards	2
		Database/schema variable mapping; ETL-based standardization; manual terminology mapping	1
		ETL-based standardization; established data standards; local data dictionary	1
		ETL-based standardization; established data standards; national/global standards	1
		Database/schema variable mapping; local data dictionary; national/global standards	1
		ETL-based standardization; local data dictionary; national/global standards	1

Studies may report multiple data harmonization methods; therefore, Panel A totals may exceed N.

Figure 3 shows the evidence map of the distribution of data element harmonization methods across various health and disease program areas as reported in the articles. Overall, established data standards and national/global standards were the most frequently reported harmonization approaches, while manual terminology mapping and local data dictionaries were least represented. There were more studies on cancer, primary care, notifiable diseases, immunization, sexually transmitted infections/diseases, and chronic disease contexts. Specialized and service-focused areas (eg, neonatal care, palliative care, elderly care, nephrology, and community-based care) had fewer studies across data element harmonization methods.

**Figure 3.**
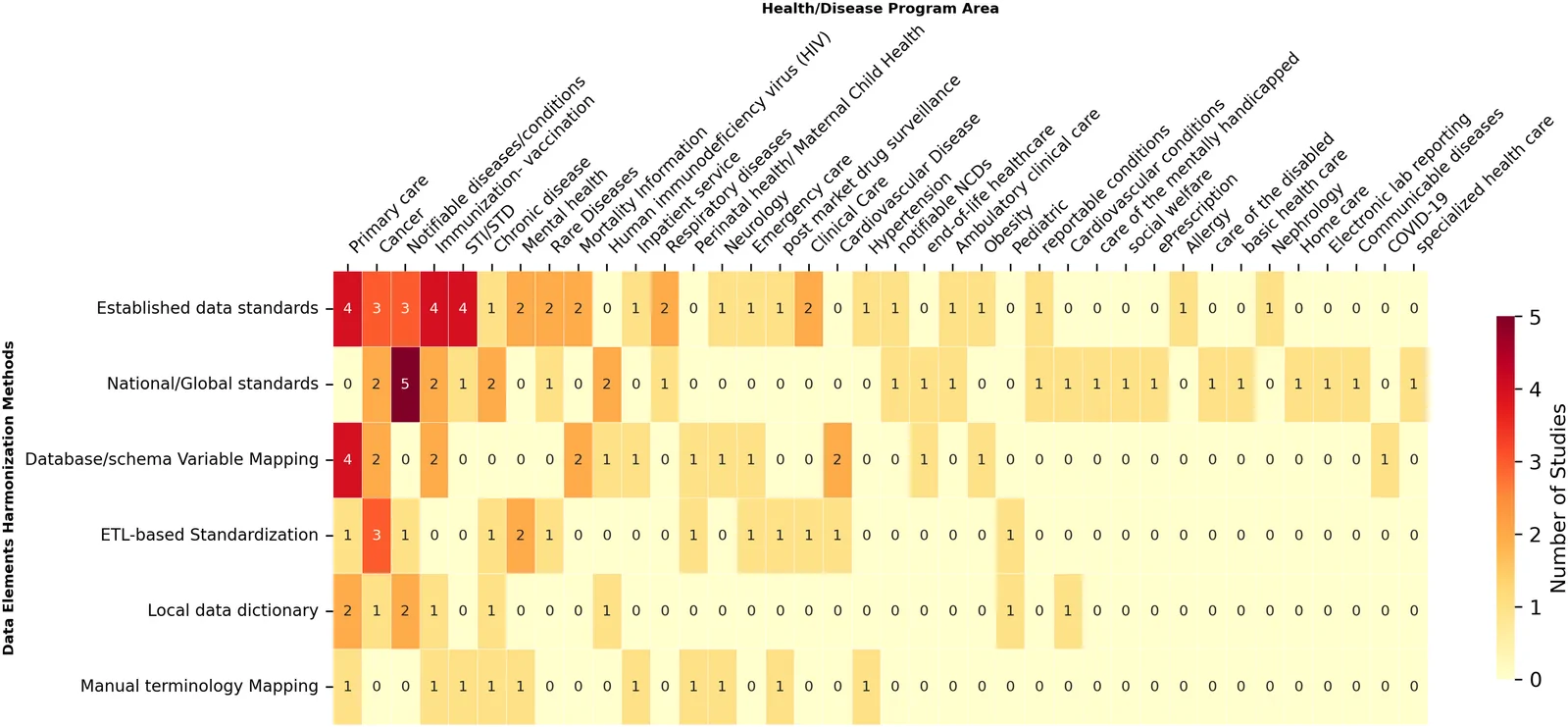
Evidence Map for the Distribution of Data Element Harmonization Methods Used in Program Areas.

### Reported Data Quality Attributes

Across 69 study articles, the most frequently reported data quality attribute was completeness (43 [62%]), followed by accuracy (29 [42%]) and timeliness (24 [35%]). Less commonly reported were accessibility (16 [23%]), validity (10 [15%]), consistency (9 [13%]), relevance (2 [3%]), and usefulness (1 [1%]), as shown in Table 4A. With respect to the number of data quality attributes reported per article, 22 (32%) articles reported 1 attribute, 29 (42%) reported 2 attributes, and 18 (26%) reported 3 attributes (Table 4B).

**Table 4.** Frequency of Data Quality Attributes Reported and Breadth of Reporting (N = 69)

**A. Frequency of data quality attributes reported**			
**Data quality attribute**			**Number of studies, n (%)**
Completeness			43 (62%)^1–4^^,^^6–8^^,^^10^^,^^11^^,^^14^^,^^20^^,^^24–28^^,^^33^^,^^35^^,^^36^^,^^38^^,^^40^^,^^41^^,^^43–45^^,^^47–50^^,^^53^^,^^54^^,^^56–62^^,^^67–69^^,^^71^^,^^74^
Accuracy			29 (42%)^3^^,^^5–8^^,^^13^^,^^15^^,^^22^^,^^24–26^^,^^31^^,^^33^^,^^34^^,^^41–47^^,^^52^^,^^53^^,^^55^^,^^57^^,^^63^^,^^65^^,^^73^^,^^75^
Timeliness			24 (35%)^2^^,^^3^^,^^8^^,^^10^^,^^18^^,^^22^^,^^40^^,^^43^^,^^45^^,^^50^^,^^52^^,^^54^^,^^57–59^^,^^61^^,^^64–66^^,^^68^^,^^70^^,^^72^^,^^73^^,^^76^
Accessibility			16 (23%)^9^^,^^10^^,^^13^^,^^16^^,^^17^^,^^31^^,^^34^^,^^36^^,^^46^^,^^48^^,^^51^^,^^56^^,^^59^^,^^69^^,^^70^^,^^73^
Validity			10 (15%)^4^^,^^18^^,^^21^^,^^24^^,^^26^^,^^36^^,^^39^^,^^41^^,^^65^^,^^75^
Consistency			9 (13%)^5^^,^^20^^,^^31^^,^^38^^,^^49^^,^^51^^,^^53^^,^^55^^,^^62^
Relevance			2 (3%)^33^^,^^48^
Usefulness			1 (1%)^54^

**B. Breadth of reporting: number of data quality attributes reported per article**			
**Attributes per article**	**No. of articles, n (%)**	**Attributes reported**	**No. of articles**
1 Attribute	22 (32%)	Completeness	10
		Timeliness	4
		Accuracy	3
		Accessibility	3
		Validity	2
2 Attributes	29 (43%)	Completeness; timeliness	6
		Accuracy; completeness	5
		Completeness; consistency	4
		Accessibility; accuracy	3
		Accuracy; consistency	2
		Accessibility; completeness	2
		Accuracy; timeliness	2
		Accessibility; timeliness	1
		Timeliness; validity	1
		Accessibility; consistency	1
		Accuracy; validity	1
		Completeness; validity	1
3 Attributes	18 (26%)	Accuracy; completeness; timeliness	5
		Accuracy; completeness; validity	3
		Accessibility; completeness; timeliness	2
		Completeness; timeliness; usefulness	1
		Accuracy; completeness; consistency	1
		Accuracy; completeness; relevance	1
		Accuracy; timeliness; validity	1
		Accessibility; completeness; relevance	1
		Accessibility; accuracy; consistency	1
		Accessibility; completeness; validity	1
		Accessibility; accuracy; timeliness	1

An attribute was counted as reported if the article explicitly stated it in relation to integration/harmonization. Studies may report multiple attributes; therefore, Panel A totals may exceed N.

An evidence map (Figure 4) visualizes data quality attributes that were commonly co-reported with particular data element harmonization methods, with data element harmonization methods on the vertical axis and data quality attributes on the horizontal axis. The number in each cell represents the number of studies that co-reported a specific data element harmonization method and data quality attribute. Established data standards were the most frequently used method and were linked to all reported data quality attributes. Use of a data dictionary was less commonly reported and associated with fewer data quality attributes.

**Figure 4.**
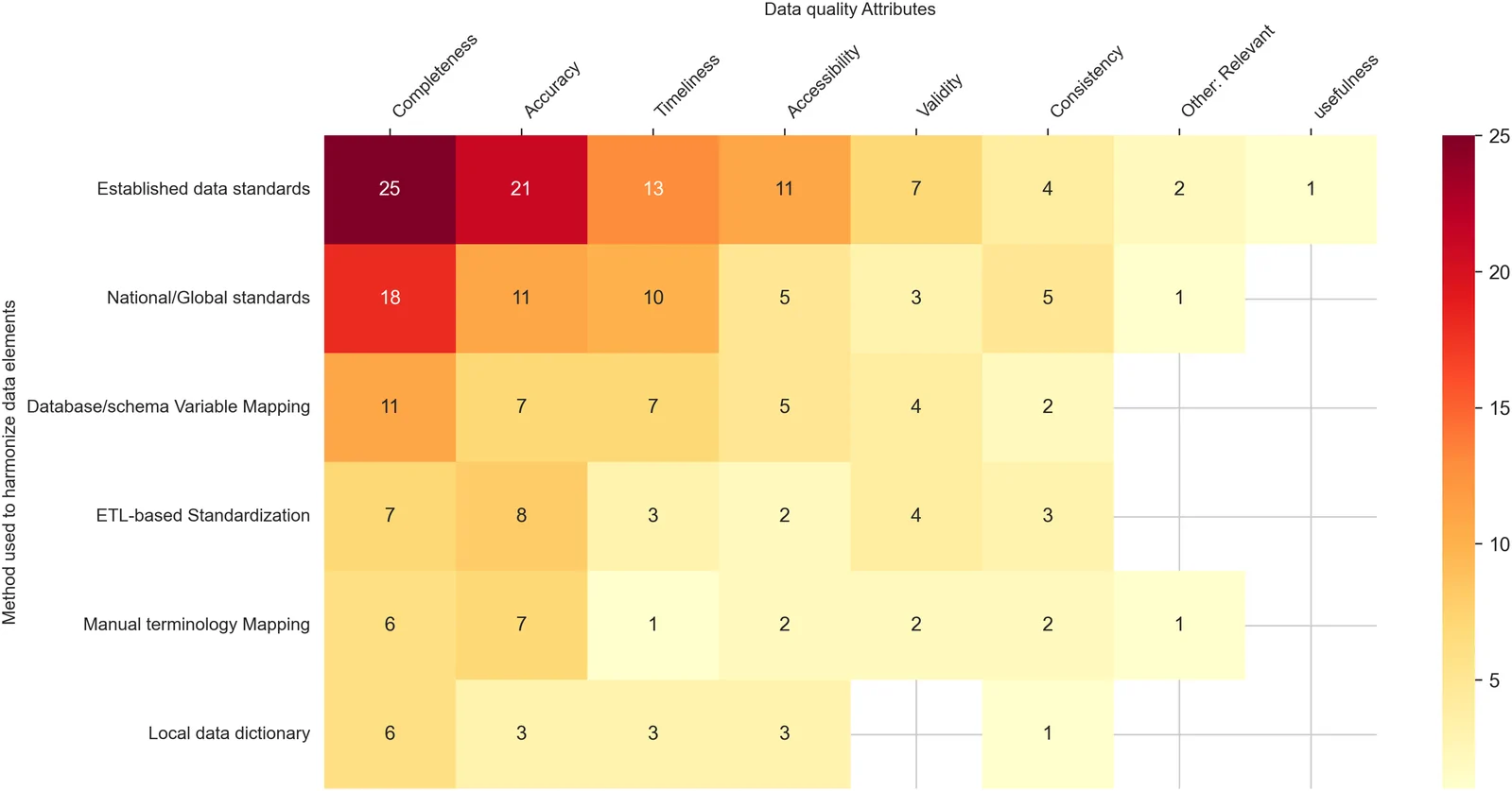
Evidence Map on Co-Reporting of Data Element Harmonization Methods and Data Quality Attributes.

### Data Element Harmonization–Reported Benefits

Table 5 summarizes reported benefits and the co-reporting context in which they appeared, including the median number of harmonization methods, data quality attributes reported, and source systems used. Of the 69 selected studies, 46 (67%) reported at least 1 benefit, with 23 (33%) not reporting. The most frequently reported benefits were data reuse for multiple purposes and reduced work burden, reported in 20 studies each, followed by improved decision-making, in 18 studies. Across benefits, studies typically reported 2 harmonization methods (range, 1-3) and 2 data quality attributes (range, 1-3), and most integrations involved 2 source systems (range, 2-5). In contrast, cost was infrequently reported (3 studies) but tended to occur in studies integrating more systems (median, 4; range, 2-4) and reporting more data quality attributes (median, 3; range, 1-3). A single study reported a new model for community-level surveillance, alongside 1 harmonization method category, 3 data quality attributes, and integration across 4 systems.

**Table 5.** Reported Benefits and the Co-reporting Context (N = 69)

**Reported benefits**	**Studies, n (%)**	**Harmonization methods, median (range)**	**Data quality attributes, median (range)**	**Systems used, median (range)**
Data reuse for multiple purposes	20 (29%)	2 (1–3)	2 (1–3)	2 (2–4)
Reduced work burden	20 (29%)	2 (1–3)	2 (1–3)	2 (2–4)
Improved decision-making	18 (26%)	2 (1–3)	2 (1–3)	2 (2–5)
Cost	3 (4%)	2 (1–2)	3 (1–3)	4 (2–4)
New model for community-level surveillance	1 (1%)	1 (1–1)	3 (3–3)	4 (4–4)

Studies may report multiple benefits, and others did not report; therefore, totals may exceed or be less than N.

Figure 5 visualizes the co-reporting of harmonization method categories and benefit categories, providing a synthesis of how reported benefits pairs with different data element harmonization methods. Data reuse, improved decision-making, and reduced work burden were the most frequently reported benefits, while cost was less commonly reported in the studies. Notably, several harmonization methods–benefits combination had no supporting studies, highlighting potential gaps.

**Figure 5.**
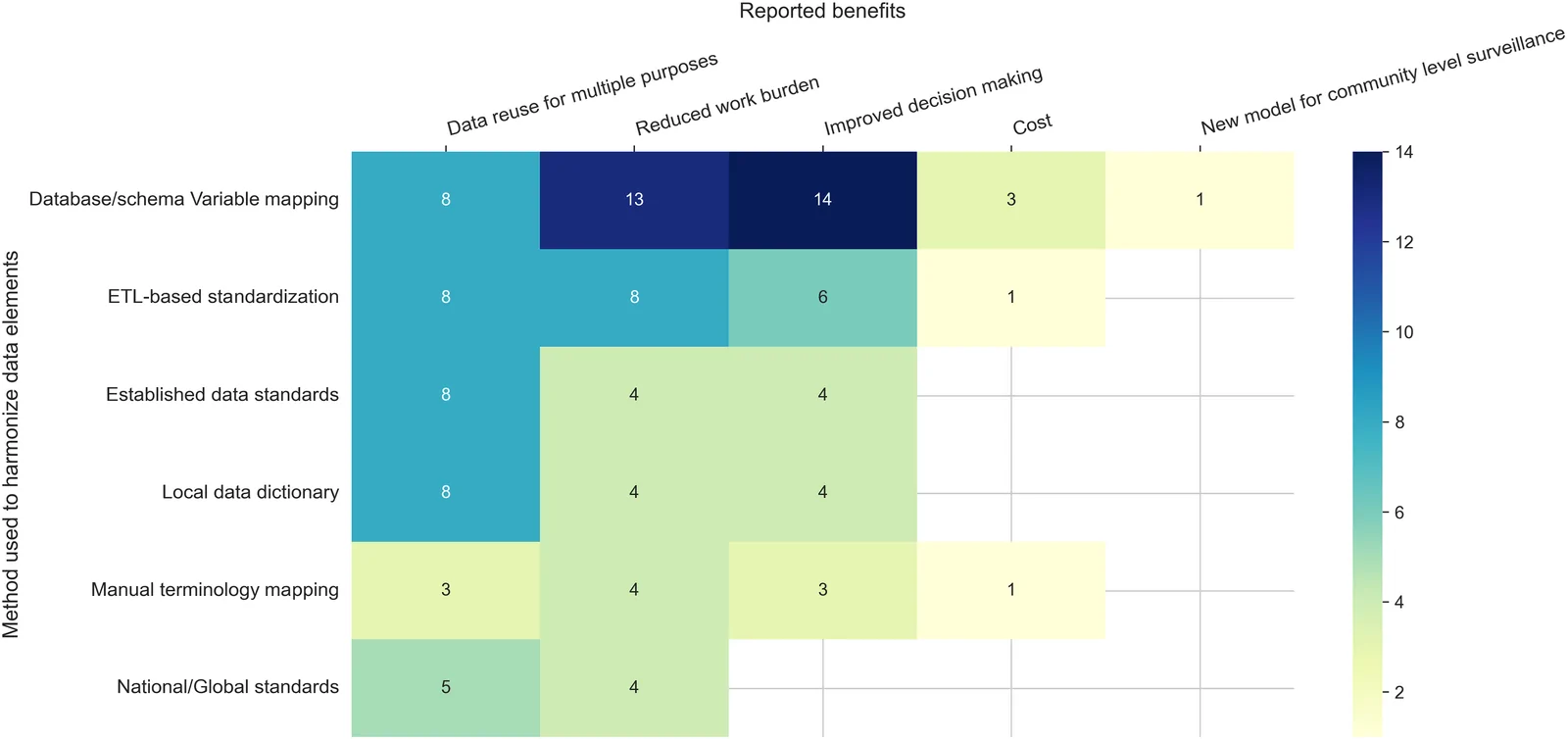
Evidence Map on Combinations of Harmonization Methods and Reported Benefits.

### Harmonization Methods Association With Number of Systems and Data Quality Attributes

Among 69 studies, 44 (63.8%) were classified as complex and 25 (36.2%) as simple (Figure 6B). This indicates that most selected studies involved more intricate combinations of methods and systems. In the complexity integration landscape, Figure 6A shows simple integrations clustered tightly at 2 systems with 1-2 methods (green-shaded region). Complex integrations were more dispersed, spanning 3-5 systems and 1-3 methods, indicating greater heterogeneity in system integration and harmonization approaches. Bubble size represents the number of data quality attributes reported, which varied across both categories, suggesting similar ranges of attribute reporting across simple and complex integrations. The landscape provides a visual summary of how studies clustered (or spread) across data integration configurations, and how the breadth of data quality attributes (number of attributes) overlaid these configurations.

**Figure 6.**
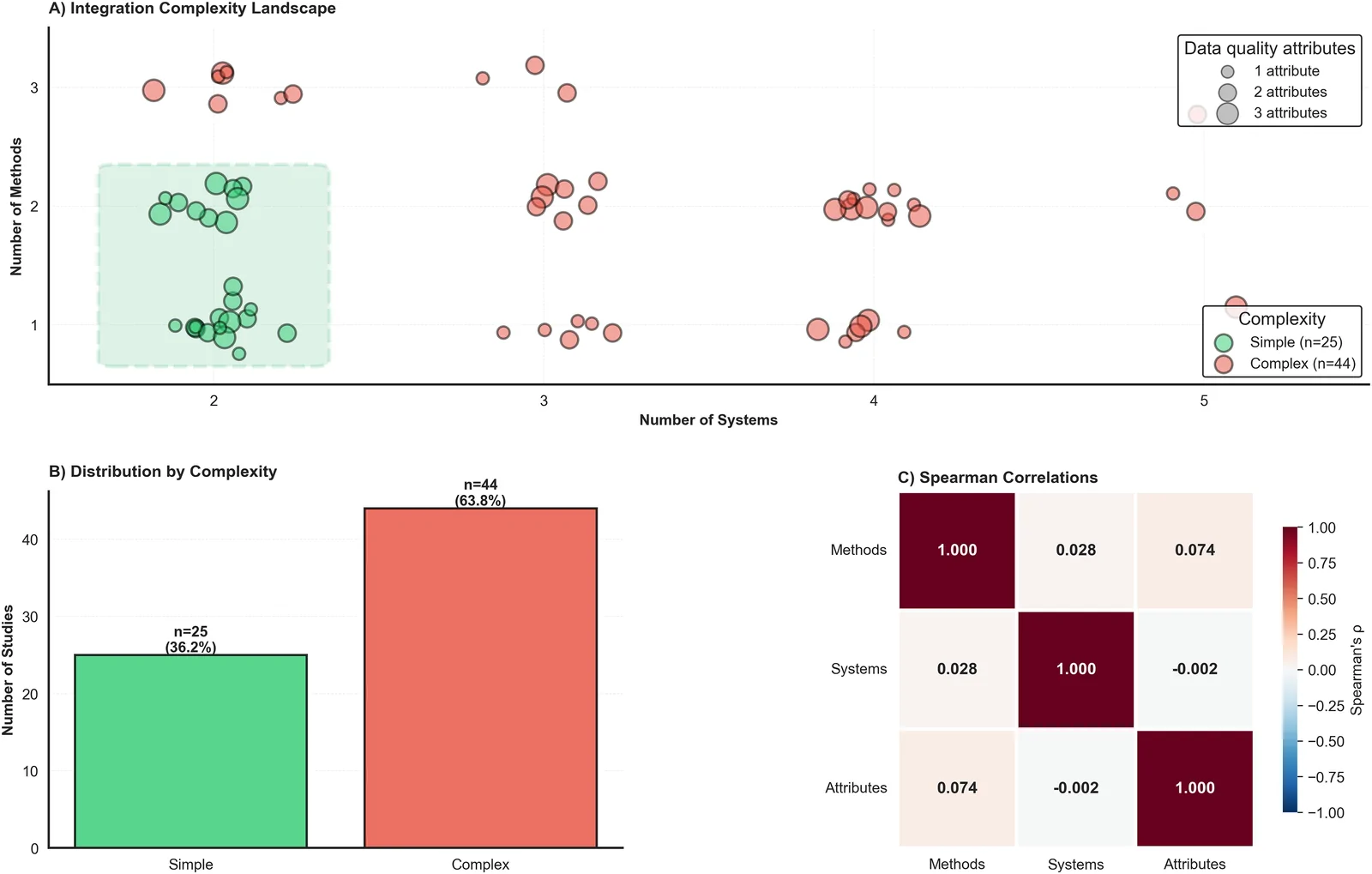
Pairwise Associations and Integration Complexity Measures Among Harmonization Methods, Number of Systems and Data Quality Attributes.

Spearman rank correlations, Figure 6C, showed near-zero associations across the 3 pairs. Associations were weak for methods vs systems (ρ = 0.028), methods vs attributes (ρ = 0.074), and systems vs attributes (ρ = −0.002).

## Discussion

The digitization of health data offers great improvements in its access and utilization, but it also has challenges related to integration from heterogeneous sources. In this review, most studies came from high-income settings, with a majority in the United States, underscoring a concentration of research in resource-rich settings. Program areas were dominated by immunization, notifiable diseases, cancer, and primary care, while many other areas appeared only sporadically. Data integration efforts primarily relied on electronic medical record systems (EMRs) and data repositories, whereas other systems such as HIEs, disease registries, and laboratory systems were less frequently incorporated. Integration typically involved 2-4 source systems, suggesting that while multisystem approaches are common, highly complex integrations remain rare. These patterns highlight both the maturity of integration practices in high-income contexts and the need for broader inclusion of diverse program areas, system types, and geographic settings to strengthen global applicability and equity in health data integration research.

This review identified 6 harmonization methods used to facilitate data integration among data source systems. One or 2 methods were commonly used to harmonize data elements before the data were integrated, whereas the use of ≥3 methods was less common, highlighting the possibility of constraints such as increased resource demand, greater implementation complexity, or both. Methods using internationally recognized and national standards were preferred and most widely utilized, reflecting their role in promoting interoperability and consistency. Approaches such as variable mapping to existing database schemas or common data models and ETL-based harmonization were moderately applied. These approaches were necessary when understanding the characteristics of data elements in each system to ensure alignment.^17^ Manual mapping and local data dictionaries, while less common, remain important in settings with limited adoption of standardized terminologies. Importantly, these data element harmonization methods are not mutually exclusive and are often applied in combination as a common practice within a single project to achieve data integration.^3^^,^^17^^,^^22^^,^^31^^,^^33^^,^^43^ Their complementarity nature supports scalable, reproducible, and semantically interoperable integration of heterogeneous data sources.

Harmonization methods were applied across diverse disease and program areas, with EMRs being the most common data source system. This reflects the broad use of electronic data sources in routine care, such as EMRs, that typically have structured data and standardized extraction processes, which provide a foundation for data integration across the health domain.^1^^,^^3^^,^^5^ Program areas with strong surveillance, registry infrastructure, or reporting mandates (eg, cancer, HIV, and immunization) tended to use standardized data harmonization approaches, reflecting established data extraction and exchange to support reporting requirements.^2^^,^^4^^,^^58^ Conversely, specialized and community-oriented areas such as neonatal care, palliative care, elderly care, and nephrology had lower representation. This is likely because of specialized workflows, limited standardization mandates, and fewer large-scale data systems.^6^^,^^23^ Some studies leveraged health information exchange (HIE) platforms to enhance clinical data. In these cases, HIE facilitated mapping local data elements to standardized vocabularies, improving data completeness compared with integrating unprocessed (raw) data directly from source systems. Access to higher-quality, standardized data ultimately supports more reliable patient-centered care and disease surveillance, benefiting both clinical and public health use.

Data completeness, accuracy, and timeliness were the most reported data quality attributes. Similar patterns have been observed in other studies, where completeness, accuracy, and timeliness were also found to be the most assessed or studied because of their centrality to basic data fitness for use.^12^^,^^26^^,^^63^^,^^77^ These findings suggest that foundational data quality attributes were prioritized to ensure that integrated data are usable, reliable, and actionable for clinical care, surveillance, and program decision‑making. Moderate reporting of accessibility, validity, and consistency suggests a growing recognition of the importance of data availability, uniformity, and accurate reflection of real-world phenomena as essential factors in decision-making. This highlights the emphasis on foundational data quality attributes for data integration. In contrast, relevance and usefulness were rarely reported, likely because these attributes are more context-specific and subjective, depending on program and user needs. Efforts to improve data quality should continue to focus on completeness and accuracy while also strengthening the processes for consistency and validity. It is also necessary to explicitly incorporate relevance and usefulness into the studies to ensure that the data element harmonization and data integration processes are context-aware and enhance data value. The most common data quality attribute combinations included completeness with timeliness or accuracy, reinforcing the emphasis on core attributes for integration.

Studies have also reported operational and strategic benefits associated with harmonization-supported data integration. Commonly cited benefits included data reuse for multiple purposes, reduced work burden, and improved decision-making. Collectively, these reported benefits emphasized reducing manual workload, enabling reuse of routinely collected data for multiple purposes, and improving the quality of information available for decision-making, underscoring the potential value for supporting data-driven insights in health programs and information systems.^12^ Cost-related considerations were less frequently reported and typically in contexts involving integration of multiple systems, indicating potential interest in financial impacts. This aligns with sociotechnical adoption dynamics, where human–technology routines are prioritized.^24^^,^^31^^,^^41^ One study reported a novel model for community-level surveillance, demonstrating the potential for harmonization to support innovative approaches in public health. Overall, these findings underscore the value of harmonization in improving data quality and enabling data-driven insights across health programs and diverse systems. However, gaps in reporting certain data quality attributes and benefits, such as relevance, usefulness, and cost, highlight opportunities to adopt comprehensive evaluation frameworks.

The analysis of integration complexity revealed distinct patterns in how harmonization methods and systems were combined across studies. Most studies reflected complex integration patterns, while simple integrations were concentrated in a narrow 2-system, 1- to 2-method configuration. This suggests that simple integrations tend to follow predictable, streamlined approaches, whereas complex integrations involve more diverse and heterogeneous strategies to accommodate multiple systems and harmonization requirements. Despite this separation between simpler and complex integration in terms of the number of systems used and the number of harmonization methods applied, the breadth of data quality attributes did not systematically increase with either more systems or more harmonization methods. This observation was reinforced by near-zero Spearman rank correlations among the 3 dimensions of the number of harmonization methods used, the number of systems involved, and the number of data quality attributes reported, indicating no meaningful monotonic relationships. These findings suggest that the integration complexity and data quality attributes reported may scale independently. Practically, this implies that increasing the number of systems for integration or adding harmonization methods does not necessarily translate into broader data quality attribute reporting. Future work should assess whether this independence reflects true differences in practice vs differences in reporting. In addition, exploring whether specific combinations of systems and harmonization strategies are associated with particular data quality attributes could provide insights for designing frameworks that balance complexity with data quality assurance. Understanding contextual factors, such as implementation strategy, resource allocation, workforce capability, organizational, and data use contexts, is key to supporting programmatic decision-making, alignment with governance, and operational improvements.^29^^,^^30^^,^^63^ These findings should be interpreted cautiously and do not imply causal relationships; rather, they highlight the need for more systematic approaches to harmonization and data quality assessment when integrating data from diverse HISs.^17^^,^^18^^,^^21^^,^^26^^,^^28^^,^^63^

There are several limitations to this systematic review that might constrain the interpretation and the strength of inferences drawn from the review analysis. First, the evidence base is heavily concentrated in high-income settings, particularly the United States, which may limit the generalizability of reported harmonization approaches and data quality assessments to low- and middle-income country contexts. Differences in HISs infrastructure, interoperability standards, data governance, and technical capacity may affect implementation and performance. Second, we did not perform a formal appraisal of methodological quality (eg, risk of bias) because the included studies were highly heterogeneous in design, context, and outcomes; consequently, our findings should be interpreted as a descriptive synthesis of reported harmonization approaches and assessed data quality attributes rather than as evidence of comparative effectiveness. Third, inter-rater agreement during study selection showed moderate to high observed agreement between reviewers; however, Cohen κ values were modest. This likely reflects chance-corrected agreement statistics because of imbalances toward 1 category, particularly in systematic review screening where the majority of studies are either excluded or included. To mitigate this limitation, reviewers completed a calibration exercise prior to formal screening to ensure consistent interpretation of eligibility criteria. In addition, all discrepancies were resolved through discussion. These procedures helped ensure consistent application of inclusion criteria despite diverse expertise and the statistical limitations of κ.^78^ Fourth, this review was not prospectively registered in a systematic review registry such as International Prospective Register of Systematic Reviews (PROSPERO), which may reduce protocol transparency; however, adherence to Preferred Reporting Items for Systematic Reviews and Meta-Analyses guidelines and a predefined analytic plan helped mitigate this risk.

## Conclusions

This systematic review summarizes published approaches to data element harmonization used to support integration of routinely collected health data across multiple HISs and programs. Six methods were identified, with established data standards being the most frequently used while manual and localized approaches remain in use, indicating a preference for standardized and automated methods in data integration and interoperability efforts. Most studies applied 1 or 2 methods to achieve harmonization. This indicates that the maturity of program infrastructure, reporting mandates, and level of data standardization in underlying data systems inform data harmonization choices. Foundational data quality attributes and sociotechnical adoption dynamics were prioritized, as indicated by data completeness, accuracy, and timeliness being the most frequently reported data quality attributes and reported benefits including data reuse, improved decision-making, and reduced work burden in the studies. There was no meaningful association found between the number of harmonization methods used and either the number of systems used for data integration or the number of data quality attributes reported. This suggests that applying more harmonization methods does not appear to be associated with measurable increase in data quality attribute outcomes or the number of systems used. Our findings should be interpreted as a descriptive synthesis of reported harmonization approaches and assessed data quality attributes rather than as evidence of comparative effectiveness. Efforts to improve data quality should continue to focus on completeness and accuracy while also strengthening processes for consistency and validity. Explicitly incorporating relevance and usefulness into data quality assessment could further enhance the value of data and its utility for health.

These findings suggest that factors beyond the sheer number of data element harmonization methods across systems may influence data integration from multiple sources and improvements in data quality. Comparing studies that utilize already collected data with those that involve standardization before data collection in real-world settings, particularly in low- and middle-income countries, can help highlight the efforts and resources needed to address most data quality issues downstream, making analysis faster, more reliable, and cost-effective. Future research should also explore contextual factors such as implementation strategy, resource allocation, workforce capability, and organizational or data use context. In addition, qualitative and comparative assessments of harmonization methods should be considered to better understand the underlying mechanisms or causal pathways that drive successful outcomes. This additional research will be important, especially in low- and middle-income countries where some global initiatives, including the United States Government global health programs, have supported digital health and interoperability implementations.

## Acknowledgments

This publication has been supported by the United States Government (USG) through the US Centers for Disease Control and Prevention (CDC).

## Author Contributions

All authors contributed to drafting, reviewing, and approving the final manuscript.

## Declaration of Competing Interest

The authors declare that they have no known competing financial interests or personal relationships that could have appeared to influence the work reported in this paper.

## Disclosures

Existing text in this manuscript was reviewed using ChatGPT version 5.2 and Microsoft 365 Copilot powered by GPT-5.2 for clarity and summarized results generated from analysis. Claude based on Sonnet version 4.5 and ChatGPT version 5.2 were used to draft python code used to generate analysis and figures in Jupyter notebooks.

The authors declare no conflicts of interest.

## Funding

The authors received no external funding for this research.

## Ethics and Data Availability

This study did not involve human subjects and therefore did not require IRB review. All data for this study were derived from publicly available published sources.

## Disclaimer

The findings and conclusions in this report are those of the authors and do not necessarily represent the official position of the US Centers for Disease Control and Prevention (CDC) and funding agencies.

CE Quiz

## Supplementary Material

Supplemental Material

## References

[1] Newton-Dame R, McVeigh KH, Schreibstein L, Design of the New York City Macroscope: innovations in population health surveillance using electronic health records. EGEMS (Wash DC). 2016;4(1):1265. doi:10.13063/2327-9214.1265. PMC5226383 28154835

[2] Troppy S, Haney G, Cocoros N, Cranston K, DeMaria A. Jr. Infectious disease surveillance in the 21st century: an integrated web-based surveillance and case management system. Public Health Rep. 2014;129(2):132-138. doi:10.1177/003335491412900206. PMC3904892 24587547

[3] Kariuki JM, Manders EJ, Richards J, Automating indicator data reporting from health facility EMR to a national aggregate data system in Kenya: an interoperability field-test using OpenMRS and DHIS2. Online J Public Health Inform. 2016;8(2):e188. doi:10.5210/ojphi.v8i2.6722. PMC5266757 28149444

[4] Aniekwe C, Cuffe K, Audu I, Assessing the effect of electronic health information exchange on the completeness and validity of data for measuring viral load testing turnaround time in Nigeria. Int J Med Inform. 2023;174:105059. doi:10.1016/j.ijmedinf.2023.105059. PMC11187829 37002987

[5] Horth RZ, Wagstaff S, Jeppson T, Use of electronic health records from a statewide health information exchange to support public health surveillance of diabetes and hypertension. BMC Public Health. 2019;19(1):1106. doi:10.1186/s12889-019-7367-z. PMC6694493 31412826

[6] Miyoshi NSB, Azevedo-Marques JM, Alves D, Azevedo-Marques PM. An eHealth Platform for the Support of a Brazilian Regional Network of Mental Health Care (eHealth-Interop): development of an interoperability platform for mental care integration. JMIR Ment Health. 2018;5(4):e10129. doi:10.2196/10129. PMC6303678 30530455

[7] D’Amore JD, McCrary LK, Denson J, Clinical data sharing improves quality measurement and patient safety. J Am Med Inform Assoc. 2021;28(7):1534-1542. doi:10.1093/jamia/ocab039. PMC8279795 33712850

[8] Asikainen P, Suominen T, Itälä T, Implementing the regional information system to integrate social and health care services – from data transfer towards effective care. Online Journal of Nursing Informatics. 2009;13(1):1-27.

[9] Smith EA, Lapinski J, Lichty-Hess J, Pier K. Using health information technology and data to improve chronic disease outcomes in Federally Qualified Health Centers in Maryland. Prev Chronic Dis. 2016;13:E178-7. doi:10.5888/pcd13.160445. PMC5201151 28033091

[10] Rajamani S, Kayser A, Ruprecht A, Electronic case reporting (eCR) of COVID-19 to public health: implementation perspectives from the Minnesota Department of Health. J Am Med Inform Assoc. 2022;29(11):1958-1966. doi:10.1093/jamia/ocac133. PMC9384568 35904765

[11] Dixon BE, Siegel JA, Oemig TV, Grannis SJ. Electronic health information quality challenges and interventions to improve public health surveillance data and practice. Public Health Rep. 2013;128(6):546-553. doi:10.1177/003335491312800614. PMC3804098 24179266

[12] Liaw ST, Rahimi A, Ray P, Towards an ontology for data quality in integrated chronic disease management: a realist review of the literature. International Journal of Medical Informatics. 2013;82(1):10-24. doi:10.1016/j.ijmedinf.2012.10.001. 23122633

[13] Laws R, Gillespie S, Puro J, The Community Health Applied Research Network (CHARN) Data Warehouse: a resource for patient-centered outcomes research and quality improvement in underserved, safety net populations. EGEMS (Wash DC). 2014;2(3):1097. doi:10.13063/2327-9214.1097. PMC4371501 25848623

[14] Bennett KJ, Mann J, Ouyang L. Utilizing combined claims and clinical datasets for research among potential cases of rare diseases. Int J Healthc Inf Syst Inform. 2018;13(2):1-12. doi:10.4018/ijhisi.2018040101. PMC7479970 32913425

[15] Ma J, Beliveau J, Snider W, Jordan W, Casarett D. Combining multiple decedent data sources for a population-based picture of end-of-life healthcare utilization. J Pain Symptom Manage. 2021;62(3):e200-e205. doi:10.1016/j.jpainsymman.2021.03.005. 33722688

[16] Pan X, Zhou X, Song H, Zhang R, Zhang T. Enhanced Data Extraction, Transforming and Loading Processing for Traditional Chinese Medicine Clinical Data Warehouse. 2012:57-61.

[17] Yoon D, Ahn EK, Park MY, Conversion and data quality assessment of electronic health record data at a Korean Tertiary Teaching Hospital to a common data model for distributed network research. Healthc Inform Res. 2016;22(1):54-58. doi:10.4258/hir.2016.22.1.54. PMC4756059 26893951

[18] Sidky H, Young JC, Girvin AT, N3C Consortium. Data quality considerations for evaluating COVID-19 treatments using real world data: learnings from the National COVID Cohort Collaborative (N3C). BMC Med Res Methodol. 2023;23(1):46. doi:10.1186/s12874-023-01839-2. PMC9936475 36800930

[19] Řezník T, Raes L, Stott A, Improving the documentation and findability of data services and repositories: a review of (meta)data management approaches. Computers & Geosciences. 2022;169:105194. doi:10.1016/j.cageo.2022.105194.

[20] SÁEz C, Moner D, GarcÍA-De-LeÓN-Chocano R, A standardized and data quality assessed maternal-child care integrated data repository for research and monitoring of best practices: a pilot project in Spain. Studies in Health Technology & Informatics. 2017;235:539-543. doi:10.3233/978-1-61499-753-5-539. 28423851

[21] Tahar K, Verbuecheln R, Martin T, Graessner H, Krefting D. Local data quality assessments on ehr-based real-world data for rare diseases. In: Caring Is Sharing – Exploiting the Value in Data for Health and Innovation: Proceedings of MIE 2023. SAGE Publications; 2023:292-296. doi:10.3233/SHTI230121. 37203665

[22] Shalhout SZ, Saqlain F, Wright K, Akinyemi O, Miller DM. Generalizable EHR-R-REDCap pipeline for a national multi-institutional rare tumor patient registry. JAMIA Open. 2022;5(1):ooab118. doi:10.1093/jamiaopen/ooab118. PMC8827011 35156001

[23] Roos M, López Martin E, Wilkinson MD. Preparing data at the source to foster interoperability across rare disease resources. In: Posada de la Paz M, Taruscio D, Groft SC, eds. Rare Diseases Epidemiology: Update and Overview. Springer International Publishing; 2017;165-179. doi:10.1007/978-3-319-67144-4_9. 29214571

[24] Botts N, Bouhaddou O, Bennett J, Data quality and interoperability challenges for eHealth Exchange participants: observations from the Department of Veterans Affairs' Virtual Lifetime Electronic Record Health pilot phase. AMIA Annu Symp Proc. 2014;2014:307-314. PMC4419918 25954333

[25] Bae CJ, Griffith S, Fan Y, The challenges of data quality evaluation in a joint data warehouse. EGEMS (Wash DC). 2015;3(1):1125. doi:10.13063/2327-9214.1125. PMC4537084 26290882

[26] Verma AA, Pasricha SV, Jung HY, Assessing the quality of clinical and administrative data extracted from hospitals: the General Medicine Inpatient Initiative (GEMINI) experience. J Am Med Inform Assoc. 2021;28(3):578-587. doi:10.1093/jamia/ocaa225. PMC7936532 33164061

[27] Priou S, Lame G, Jankovic M, Why Are Data Missing in Clinical Data Warehouses? A simulation study of how data are processed (and can be lost). Stud Health Technol Inform. 2023;302:202-206. doi:10.3233/SHTI230103. 37203647

[28] Thuraisingam S, Chondros P, Dowsey MM, Assessing the suitability of general practice electronic health records for clinical prediction model development: a data quality assessment. BMC Med Inform Decis Mak. 2021;21(1):297-211. doi:10.1186/s12911-021-01669-6. PMC8557028 34717599

[29] Hosseinzadeh E, Afkanpour M, Momeni M, Tabesh H. Data quality assessment in healthcare, dimensions, methods and tools: a systematic review. BMC Med Inform Decis Mak. 2025;25(1):296. doi:10.1186/s12911-025-03136-y. PMC12335082 40783704

[30] Lewis AE, Weiskopf N, Abrams ZB, Electronic health record data quality assessment and tools: a systematic review. J Am Med Inform Assoc. 2023;30(10):1730-1740. doi:10.1093/jamia/ocad120. PMC10531113 37390812

[31] Garies S, Cummings M, Forst B, Achieving quality primary care data: a description of the Canadian Primary Care Sentinel Surveillance Network data capture, extraction, and processing in Alberta. Int J Popul Data Sci. 2019;4(2):1132. doi:10.23889/ijpds.v4i2.1132. PMC8142949 34095540

[32] Landis JR, Koch GG. The measurement of observer agreement for categorical data. Biometrics. 1977;33(1):159-174. 843571

[33] El Fadly A, Rance B, Lucas N, Integrating clinical research with the Healthcare Enterprise: from the RE-USE project to the EHR4CR platform. J Biomed Inform. 2011;44 Suppl 1:S94-S102. doi:10.1016/j.jbi.2011.07.007. 21888989

[34] Dixon BE, Apathy NC. Interoperability in the Wild: comparison of Real-World Electronic C-CDA Documents from Two Sources… 19th World Congress on Medical and Health Informatics, July 8-12, 2023, Sydney, New South Wales. Medinfo. 2023;310:43-47. doi:10.3233/SHTI230924. 38269762

[35] Todd JV, Collins NV, Oakley J, Automating case reporting of chlamydia and gonorrhea to public health authorities in Oregon clinics. Sex Transm Dis. 2022;49(1):38-42. doi:10.1097/OLQ.0000000000001507. PMC10258727 34618780

[36] Bailey LC, Milov DE, Kelleher K, Multi-institutional sharing of electronic health record data to assess childhood obesity. PLoS One. 2013;8(6):e66192. doi:10.1371/journal.pone.0066192. PMC3688837 23823186

[37] Bank W. Data from: World Bank Country and Lending Groups: Historical Country Classification (Dataset CLASS_2025_07_02). World Development Indicators. 2025 Washington, DC.

[38] Bian J, Loiacono A, Sura A, Implementing a hash-based privacy-preserving record linkage tool in the OneFlorida clinical research network. JAMIA Open. 2019;2(4):562-569. doi:10.1093/jamiaopen/ooz050. PMC6994009 32025654

[39] Curtis MD, Griffith SD, Tucker M, Development and validation of a high-quality composite real-world mortality endpoint. Health Serv Res. 2018;53(6):4460-4476. doi:10.1111/1475-6773.12872. PMC6232402 29756355

[40] Groom HC, Crane B, Naleway AL, Monitoring vaccine safety using the vaccine safety Datalink: assessing capacity to integrate data from Immunization Information systems. Vaccine. 2022;40(5):752-756. doi:10.1016/j.vaccine.2021.12.048. PMC8719644 34980508

[41] Hawley S, Yu J, Bogetic N, Digitization of measurement-based care pathways in mental health through REDCap and electronic health record integration: development and usability study. J Med Internet Res. 2021;23(5):e25656. doi:10.2196/25656. PMC8176343 34014169

[42] Hosking K, Stewart G, Mobsby M, Data linkage and computerised algorithmic coding to enhance individual clinical care for aboriginal people living with chronic hepatitis B in the Northern Territory of Australia - Is it feasible? PLoS One. 2020;15(4):e0232207. doi:10.1371/journal.pone.0232207. PMC7188233 32343712

[43] Huang H, Ng MY, Wu JT, Automating the renal cell carcinoma registry in Singapore: a case study on the integration of the research electronic data capture system with the enterprise data warehouse. J Registry Manag. 2018;45(4):156-160. 31490910

[44] Koepke R, Petit AB, Ayele RA, Completeness and accuracy of the Wisconsin immunization registry: an evaluation coinciding with the beginning of meaningful use. J Public Health Manag Pract. 2015;21(3):273-281. doi:10.1097/PHH.0000000000000216. 25590511

[45] Li S, Zhang L, Liu S, Hubbard R, Li H. Surveillance of noncommunicable disease epidemic through the integrated noncommunicable disease collaborative management system: feasibility pilot study conducted in the city of Ningbo, China. J Med Internet Res. 2020;22(7):e17340. doi:10.2196/17340. PMC7413277 32706706

[46] Matoba T, Kohro T, Fujita H, Architecture of the Japan Ischemic heart disease Multimodal Prospective data Acquisition for preCision Treatment (J-IMPACT) System. Int Heart J. 2019;60(2):264-270. doi:10.1536/ihj.18-113. 30799376

[47] Mishra NK, Jellison JB, Hamilton A, Carr JB, Padilla RM, Viator NA. Leveraging informatics to identify reportable cases: pilot findings on electronic case reporting of chlamydia and gonorrhea. J Public Health Manag Pract. 2019;25(6):595-597. doi:10.1097/PHH.0000000000000954. 30789599

[48] Mishra N, Duke J, Karki S, A modified public health automated case event reporting platform for enhancing electronic laboratory reports with clinical data: design and implementation study. J Med Internet Res. 2021;23(8):e26388. doi:10.2196/26388. PMC8387889 34383669

[49] Rajamani S, Kayser A, Emerson E, Solarz S. Evaluation of data exchange process for interoperability and impact on electronic laboratory reporting quality to a state public health agency. Online J Public Health Inform. 2018;10(2):e204. doi:10.5210/ojphi.v10i2.9317. PMC6194099 30349622

[50] Ranade-Kharkar P, Pollock SE, Mann DK, Thornton SN. Improving clinical data integrity by using data adjudication techniques for data received through a health information exchange (HIE). AMIA Annu Symp Proc. 2014;2014:1894-1901. PMC4419943 25954462

[51] Rayner J, Khan T, Chan C, Wu C. Illustrating the patient journey through the care continuum: leveraging structured primary care electronic medical record (EMR) data in Ontario, Canada using chronic obstructive pulmonary disease as a case study. Int J Med Inform. 2020;140:104159. doi:10.1016/j.ijmedinf.2020.104159. 32473567

[52] Rutzner S, Fietkau R, Ganslandt T, Prokosch HU, Lubgan D. Electronic support for retrospective analysis in the field of radiation oncology: proof of principle using an example of fractionated stereotactic radiotherapy of 251 meningioma patients. Front Oncol. 2017;7:16. doi:10.3389/fonc.2017.00016. PMC5298960 28232905

[53] Rutzner S, Ganslandt T, Fietkau R, Prokosch HU, Lubgan D. Noncurated data lead to misinterpretation of treatment outcomes in patients with prostate cancer after salvage or palliative radiotherapy. JCO Clin Cancer Inform. 2019;3:1-11. doi:10.1200/CCI.19.00052. 31599645

[54] Al-Samarrai T, Wu W, Begier E, Evaluation of a pilot respiratory virus surveillance system linking electronic health record and diagnostic data. J Public Health Manag Pract. 2013;19(4):322-329. doi:10.1097/PHH.0b013e3182602ef6. 23449125

[55] Baca CM, Benish S, Videnovic A, Axon Registry® data validation: accuracy assessment of data extraction and measure specification. Neurology. 2019;92(18):847-858. doi:10.1212/WNL.0000000000007404. 30952797

[56] Byrne CM, Mercincavage LM, Bouhaddou O, The Department of Veterans Affairs' (VA) implementation of the Virtual Lifetime Electronic Record (VLER): findings and lessons learned from Health Information Exchange at 12 sites. Int J Med Inform. 2014;83(8):537-547. doi:10.1016/j.ijmedinf.2014.04.005. 24845146

[57] Dixon BE, Taylor DE, Choi M, Riley M, Schneider T, Duke J. Integration of FHIR to facilitate electronic case reporting: results from a pilot study. Stud Health Technol Inform. 2019;264:940-944. doi:10.3233/SHTI190362. 31438062

[58] Dixon BE, Zhang Z, Arno JN, Revere D, Joseph Gibson P, Grannis SJ. Improving notifiable disease case reporting through electronic information exchange–facilitated decision support: a controlled before-and-after trial. Public Health Rep. 2020;135(3):401-410. doi:10.1177/0033354920914318. PMC7238703 32250707

[59] Effler P, Ching-Lee M, Bogard A, Statewide system of electronic notifiable disease reporting from clinical laboratories: comparing automated reporting with conventional methods. JAMA: Journal of the American Medical Association. 1999;282(19):1845-1850. doi:10.1001/jama.282.19.1845. 10573276

[60] Eitelhuber T, Ngeh S, Bloomfield L, Chandaria B, Effler P. Using data linkage to monitor COVID-19 vaccination: development of a vaccination linked data repository. Int J Popul Data Sci. 2020;5(4):1730. doi:10.23889/ijpds.v5i4.1730. PMC10464866 37649990

[61] Gibney KB, Cheng AC, Hall R, Leder K. Australia's National Notifiable Diseases Surveillance System 1991-2011: expanding, adapting and improving. Epidemiol Infect. 2017;145(5):1006-1017. doi:10.1017/S0950268816002752. PMC9507803 28065205

[62] Hendrickson BK, Panchanathan SS, Petitti D. Evaluation of immunization data completeness within a large community health care system exchanging data with a state immunization information system. J Public Health Manag Pract. 2015;21(3):288-295. doi:10.1097/PHH.0000000000000045. 24378608

[63] Khare R, Utidjian L, Ruth BJ, A longitudinal analysis of data quality in a large pediatric data research network. J Am Med Inform Assoc. 2017;24(6):1072-1079. doi:10.1093/jamia/ocx033. PMC6259665 28398525

[64] Langabeer JR, Ii, Champagne-Langabeer T, Smith D. Quality improvement for cardiovascular care developing a rural, community-based registry for cardiovascular quality improvement. Qual Manag Health Care. 2018;27(4):209-214. doi:10.1097/QMH.0000000000000189. 30260928

[65] Ohlund SE, Astrand B, Petersson G. Improving interoperability in eprescribing. Interact J Med Res. 2012;1(2):e17. doi:10.2196/ijmr.2089. PMC3626130 23612314

[66] Petrides AK, Bixho I, Goonan EM, Goonan EMMSMTSHRN. The benefits and challenges of an interfaced electronic health record and laboratory information system: effects on laboratory processes. Arch Pathol Lab Med. 2017;141(3):410-417. doi:10.5858/arpa.2016-0146-OA. 28234574

[67] Pitts SI, Maruthur NM, Wang X, Team-based health information exchange use increased mammography documentation and referral in an academic primary care practice: an interrupted time series. J Gen Intern Med. 2018;33(5):710-714. doi:10.1007/s11606-017-4259-8. PMC5910338 29392596

[68] Schauer SL, Maerz TR, Verdon MJ, Hopfensperger DJ, Davis JP. The Wisconsin immunization registry experience: comparing real-time and batched file submissions from health care providers. WMJ. 2014;113(3):102-106. 25118438

[69] Schmidt D, Osmanodja B, Pfefferkorn M, TBase - an integrated electronic health record and research database for kidney transplant recipients. J Vis Exp. 2021;(170). doi:10.3791/61971. 33938875

[70] Scott HF, Brilli RJ, Paul R, Improving Pediatric Sepsis Outcomes (IPSO) Collaborative Investigators. Evaluating pediatric sepsis definitions designed for electronic health record extraction and multicenter quality improvement. Crit Care Med. 2020;48(10):e916-e926. doi:10.1097/CCM.0000000000004505. PMC7677030 32931197

[71] Westra BL, Oancea C, Savik K, Marek KD. The feasibility of integrating the Omaha system data across home care agencies and vendors. Comput Inform Nurs. 2010;28(3):162-171. doi:10.1097/NCN.0b013e3181d7812c. PMC3090219 20431359

[72] Woinarowicz M, Howell M. The impact of electronic health record (EHR) interoperability on immunization information system (IIS) data quality. Online J Public Health Inform. 2016;8(2):e184. doi:10.5210/ojphi.v8i2.6380. PMC5065519 27752294

[73] Zimmerman CR, Chaffee BW, Lazarou J, Maintaining the enterprise wide continuity and interoperability of patient allergy data. Am J Health Syst Pharm. 2009;66(7):671-679. doi:10.2146/ajhp070645. 19299376

[74] Dixon BE, McGowan JJ, Grannis SJ. Electronic laboratory data quality and the value of a health information exchange to support public health reporting processes. AMIA Annu Symp Proc. 2011;2011:322-330. PMC3243173 22195084

[75] Klann JG, Pfiffner PB, Natter MD, Supporting multi-sourced medication information in i2b2. AMIA Annu Symp Proc. 2015;2015:747-755. PMC4765563 26958210

[76] Lin MP, Sharma D, Venkatesh A, The Clinical Emergency Data Registry: structure, use, and limitations for research. Ann Emerg Med. 2024;83(5):467-476. doi:10.1016/j.annemergmed.2023.12.014. PMC11627304 38276937

[77] Ghalavand H, Shirshahi S, Rahimi A, Zarrinabadi Z, Amani F. Common data quality elements for health information systems: a systematic review. BMC Med Inform Decis Mak. 2024;24(1):243. doi:10.1186/s12911-024-02644-7. PMC11367888 39223578

[78] Byrt T, Bishop J, Carlin JB. Bias, prevalence and kappa. J Clin Epidemiol. May 1993;46(5):423-429. doi:10.1016/0895-4356(93)90018-v. 8501467

