# Supplementary material for: Data Harmonization Methods and Data Quality Assessment for Integrating Routinely Collected Data Across Multiple Sources: A Systematic Review
Source: Adv Health Inf Sci Pract. 2026 Oct 21;2(2):eah.000000007. doi: 10.63116/AH.000000007 (PMC13684885; doi:10.63116/AH.000000007)
Supplement: Supplementary file 1 [file ahisp-2-2-26023_Supplemental_Materials.docx]

**Data Harmonization Methods and Data Quality Assessment for Integrating Routinely Collected Data Across Multiple Sources: A Systematic Review**

## Supplement 1: Database Search Details

| **Database** | **Search string** | **# of items** |
| --- | --- | --- |
| **Search Date: February 7, 2024** | | |
| Pubmed | ((((((((("data integration"[All Fields]) OR ("interoperability"[All Fields])) OR ("health information exchange"[All Fields])) OR ("clinical data shar*"[All Fields])) OR ("data exchange"[All Fields])) OR ("information exchange"[All Fields])) OR ("electronic report*"[All Fields])) OR ("data aggregation"[All Fields])) OR (((((((metadata) OR ("standardized terminolog*"[All Fields])) OR ("clinical concept*"[All Fields])) OR ("indicator definition*")) OR ("metadata management"[All Fields])) OR ("data standard*"[All Fields])) OR ("core data element*"[All Fields])) OR ("quality measure*"[All Fields])) OR (dataset*) AND ((((((((((("electronic medical record*"[All Fields]) OR ("emr"[All Fields])) OR ("digital health"[All Fields])) OR ("ehealth"[All Fields])) OR ("health management information system*"[All Fields])) OR ("laboratory information system*"[All Fields])) OR ("lis"[All Fields])) OR ("pharmacy information systems"[All Fields])) OR ("electronic health record*"[All Fields])) OR ("electronic data"[All Fields])) OR ("computerized clinical record*"[All Fields])) OR ("data repositor*"[All Fields]) OR ("data warehouse*"[All Fields]) AND (((((("data quality"[All Fields]) OR ("information quality"[All Fields])) OR ("data completeness"[All Fields])) OR ("data accuracy"[All Fields])) OR ("data consistency"[All Fields])) OR ("data timeliness"[All Fields])) AND (english[Filter]) | 613 |
| Scopus | ( ( ( TITLE-ABS-KEY ( "Data integration" ) OR TITLE-ABS-KEY ( interoperability ) OR TITLE-ABS-KEY ( "health information exchange" ) OR TITLE-ABS-KEY ( "data exchange" ) OR TITLE-ABS-KEY ( "electronic reporting" ) OR TITLE-ABS-KEY ( "automated data sharing" ) OR TITLE-ABS-KEY ( "clinical data sharing" ) ) ) OR ( ( TITLE-ABS-KEY ( metadata ) OR TITLE-ABS-KEY ( "standardized terminolog*" ) OR TITLE-ABS-KEY ( "data standards" ) OR TITLE-ABS-KEY ( "metadata management" ) OR TITLE-ABS-KEY ( "clinical concepts" ) OR TITLE-ABS-KEY ( "indicator definitions" ) OR TITLE-ABS-KEY ( "quality measures" ) OR TITLE-ABS-KEY ( "data standardization" ) ) ) ) AND ( ( TITLE-ABS-KEY ( "digital health" ) OR TITLE-ABS-KEY ( "electronic medical records" ) OR TITLE-ABS-KEY ( emr ) OR TITLE-ABS-KEY ( "health management information systems" ) OR TITLE-ABS-KEY ( hmis ) OR TITLE-ABS-KEY ( "laboratory information systems" ) OR TITLE-ABS-KEY ( lis ) OR TITLE-ABS-KEY ( "pharmacy information systems" ) OR TITLE-ABS-KEY ( "electronic data" ) OR TITLE-ABS-KEY ( "electronic health records" ) OR TITLE-ABS-KEY ( ehr ) OR TITLE-ABS-KEY ( "computerized clinical records" ) OR TITLE-ABS-KEY ( "data repository" ) OR TITLE-ABS-KEY ( "data warehouse" ) ) ) AND ( ( TITLE-ABS-KEY ( "Data quality" ) OR TITLE-ABS-KEY ( "data completeness" ) OR TITLE-ABS-KEY ( "Data accuracy" ) OR TITLE-ABS-KEY ( "Data consistency" ) OR TITLE-ABS-KEY ( "Data timeliness" ) OR TITLE-ABS-KEY ( "information quality" ) ) ) AND PUBYEAR > 1999 AND PUBYEAR < 2025 AND ( LIMIT-TO ( SUBJAREA , "MEDI" ) OR LIMIT-TO ( SUBJAREA , "HEAL" ) OR LIMIT-TO ( SUBJAREA , "NURS" ) OR LIMIT-TO ( SUBJAREA , "DENT" ) OR LIMIT-TO ( SUBJAREA , "IMMU" ) ) AND ( LIMIT-TO ( DOCTYPE , "ar" ) OR LIMIT-TO ( DOCTYPE , "cp" ) ) AND ( LIMIT-TO ( LANGUAGE , "English" ) ) | 279 |
| CINAHL | S1 AND S2 AND S3 AND English  S1: Data integration OR interoperability OR health information exchange OR data exchange OR electronic report* OR automatic exchange of information OR metadata OR standardized terminolog* OR metadata management OR clinical concept* OR health indicator* OR data standard* OR clinical quality measure*  S2: digital health OR ehealth OR ( electronic health record* or electronic medical record* or emr or ehr ) OR health management information system* OR laboratory information system* OR pharmacy information system* OR electronic data OR computerized patients record* OR data repositor* OR data warehouse*  S3: data quality OR data completeness OR data accuracy OR data consistency OR data timeliness OR information quality | 707 |
| ProQuest | ((("Data Integration" OR Interoperability OR "Health Information Exchange" OR "data exchange" OR "electronic report*" OR "data sharing" OR "clinical data exchange") OR (Metadata OR "Standardized terminolog*" OR "Metadata management" OR "Clinical concept*" OR "Monitoring and evaluation Indicator*" OR "Data standard*" OR "Clinical quality measure*" OR "Indicator definition*" OR "Health Indicator*")) AND ("digital health" OR ("electronic health record*" OR "electronic medical record*" OR "electronic patient record*" OR ehr OR emr) OR ("Health Management Information System*" OR "HMIS") OR ("laboratory information system*" OR "Laboratory Information Management System*" OR LIS) OR ("pharmacy information system*" OR "clinical pharmacy information system*") OR "electronic data" OR "computerized patient record*" OR ("data repository" OR "data repositories" OR "clinical data repositories") OR "data ware house*") AND ("data quality" OR "data completeness" OR ("data accuracy" OR "accurate data") OR "data consistency" OR "data timeliness" OR ("quality information" OR "quality information"))) NOT (at.exact("Feature" OR "Literature Review" OR "Commentary" OR "Editorial" OR "News" OR "Correspondence" OR "Front Page/Cover Story" OR "Letter to the Editor" OR "Audio/Video Clip" OR "Book" OR "Instructional Material/Guideline") AND la.exact("ENG") AND stype.exact("Scholarly Journals" OR "Working Papers"))  **Limits applied:**  Databases: 7 databases searched   - Computer Science Database - Consumer Health Database - Coronavirus Research Database - Health & Medical Collection - Healthcare Administration Database - Nursing & Allied Health Database - Public Health Database   Source type: Conference Papers & Proceedings, Scholarly Journals, Working Papers | 405 |

## Supplement 2: Data extraction template

| **Category** | **Attribute** | **Values/options** |
| --- | --- | --- |
| Study details | Study Id |  |
|  | Title |  |
|  | Country |  |
|  | Year of Publication |  |
|  | Study start date |  |
|  | Study end date |  |
|  | Study sources | Peer review journal article, conference proceeding |
|  | Sponsorship source | Funding source |
|  | Study identifier(s) | DOI |
|  | Comments |  |
| Corresponding author contact details | Name |  |
|  | Institution |  |
|  | Email |  |
|  | Address |  |
| Methods | Study design | Randomized controlled trial, non-randomized experimental study, Cohort study, Cross sectional study, Case control study, Qualitative research, Case series, Case report, other |
|  | Study aim |  |
|  | Study methods |  |
|  | Study Setting/environment |  |
| Population | Disease program Area |  |
|  | Types of data integrated | - Individual level data - aggregate/ indicator data |
|  | Types of systems included in the integration | - Electronic medical records/Electronic health records (EMR/EHR) - Laboratory information system (LIS) - Data repository (NDR, CDR, DWH, SHR etc.) - Disease registry - Pharmacy information system (PIS) - HMIS - Health Information Exchange (HIE) - Other |
|  | Data source system details | Data source system name  Data system type  Data source system implementation environment   - site/health facilities, above site level   Data format/ standards used for integration |
|  | Data consumer system details | Data consumer system name  Data system type  Data source system implementation environment   - site/health facilities, above site level   Data format/ standards used for integration   - Data format, data definitions, vocabulary standards |
| Intervention | Method used to standardize data elements for data integration | - Harmonized data elements by manually mapping data elements from the systems to a standardized code - Harmonized data using ETL to standardize data elements and possible values between systems. - Harmonized data elements by mapping to established national or global standards - Utilized established data standards such as ICD, LOINC, SNOMED, HL7 FHIR - Mapped data elements to consuming system database table variables - Utilized a data dictionary with locally defined data elements - Other |
| Outcomes | Primary outcome: data quality attributes | - Completeness - Accuracy - Timeliness - Accessibility - Validity - Consistency - Other |
|  | Secondary outcome: other improvements or benefits | - Efficiency - Cost - Reduced work burden - Improved decision making - Data reuse for multiple purposes - Other |

## Supplement 3: Inter-rater Reliability for Screening and Full-Text Review

| **Stage** | **Pair** | **Agree Yes** | **A Yes, B No** | **A No, B Yes** | **Agree No** | **Prop. Agreement** | **Yes Prob.** | **No Prob.** | **Random Agreement** | **Cohen’s κ** | Interpretation* |
| --- | --- | --- | --- | --- | --- | --- | --- | --- | --- | --- | --- |
| Screening | 1 | 23 | 20 | 44 | 211 | 0.78523 | 0.03244 | 0.66331 | 0.69576 | 0.29410 | Fair |
|  | 2 | 13 | 25 | 25 | 561 | 0.91987 | 0.00371 | 0.88191 | 0.88562 | 0.29944 | Fair |
|  | 3 | 11 | 9 | 10 | 28 | 0.67241 | 0.12485 | 0.41795 | 0.54281 | 0.28349 | Fair |
|  | 4 | 18 | 17 | 27 | 238 | 0.85333 | 0.01750 | 0.75083 | 0.76833 | 0.36691 | Fair |
|  | 5 | 33 | 21 | 31 | 129 | 0.75701 | 0.07547 | 0.52406 | 0.59953 | 0.39324 | Fair |
|  | 6 | 2 | 2 | 2 | 1 | 0.42857 | 0.32653 | 0.18367 | 0.51020 | -0.16667 | Poor |
| Full text | 1 | 9 | 1 | 12 | 19 | 0.68293 | 0.12493 | 0.36883 | 0.49375 | 0.37368 | Fair |
|  | 2 | 14 | 14 | 8 | 29 | 0.66154 | 0.14580 | 0.37657 | 0.52237 | 0.29138 | Fair |
|  | 3 | 3 | 2 | 5 | 5 | 0.53333 | 0.17778 | 0.31111 | 0.48889 | 0.08696 | Slight |
|  | 4 | 11 | 6 | 8 | 9 | 0.58824 | 0.27941 | 0.22059 | 0.50000 | 0.17647 | Slight |
|  | 5 | 5 | 3 | 2 | 6 | 0.68750 | 0.21875 | 0.28125 | 0.50000 | 0.37500 | Fair |

*Interpretation based on Landis and Koch (1977)
